# Efficient and stable CRISPR/Cas9-mediated genome-editing of human type 2 innate lymphoid cells

**DOI:** 10.3389/fimmu.2023.1275413

**Published:** 2023-10-05

**Authors:** Johanne Audouze-Chaud, Jessica A. Mathews, Sarah Q. Crome

**Affiliations:** ^1^ Department of Immunology, Temerty Faculty of Medicine, University of Toronto, Toronto, ON, Canada; ^2^ Toronto General Hospital Research Institute, Ajmera Transplant Centre, University Health Network, Toronto, ON, Canada

**Keywords:** innate lymphoid cells, ILC2s, natural killer cells, CRISPR/Cas9, nucleofection, IL-4, knockout, genome editing

## Abstract

Innate lymphoid cells (ILCs) are a family of innate lymphocytes with important roles in immune response coordination and maintenance of tissue homeostasis. The ILC family includes group 1 (ILC1s), group 2 (ILC2s) and group 3 (ILC3s) ‘helper’ ILCs, as well as cytotoxic Natural Killer (NK) cells. Study of helper ILCs in humans presents several challenges, including their low proportions in peripheral blood or needing access to rare samples to study tissue resident ILC populations. In addition, the lack of established protocols harnessing genetic manipulation platforms has limited the ability to explore molecular mechanism regulating human helper ILC biology. CRISPR/Cas9 is an efficient genome editing tool that enables the knockout of genes of interest, and is commonly used to study molecular regulation of many immune cell types. Here, we developed methods to efficiently knockout genes of interest in human ILC2s. We discuss challenges and lessons learned from our CRISPR/Cas9 gene editing optimizations using a nucleofection transfection approach and test a range of conditions and nucleofection settings to obtain a protocol that achieves effective and stable gene knockout while maintaining optimal cell viability. Using IL-4 as a representative target, we compare different ribonucleoprotein configurations, as well as assess effects of length of time in culture and other parameters that impact CRISPR/Cas9 transfection efficiency. Collectively, we detail a CRISPR/Cas9 protocol for efficient genetic knockout to aid in studying molecular mechanism regulating human ILC2s.

## Introduction

Innate Lymphoid Cells (ILCs) are a family of innate lymphocytes with important roles in host defense, as well as immune and tissue homeostasis (1–5). Cytotoxic Natural Killer (NK) cells produce Interferon-γ (IFN-γ) and Tumor Necrosis Factor-α (TNF-α) and are defined by co-expression of T-Box Transcription Factor (TBET) and Eomesodermin (EOMES) (1, 3). In humans they are further classified as CD56^dim^ which express antibody dependent cellular cytotoxicity (ADCC)-mediating receptor CD16, or as CD56^bright^ NK cells that exhibit low or no cytotoxicity, but instead are potent cytokine producers. NK cells have established roles in host defense against intracellular parasites as well as in antitumor immunity (3, 6). So called “helper” ILCs, including group 1 (ILC1s), group 2 (ILC2s) and group 3 (ILC3s) ILCs are not cytotoxic, and are classified based on their transcription factor and cytokine expression profiles (1, 7). ILC1s express TBET but not EOMES, secrete TNF-α and IFN-γ and participate in host defense against viruses and intracellular bacteria (1, 8, 9). ILC2s express the GATA binding protein 3 (GATA3) and produce IL-4 (in humans), IL-5, IL-9 and IL-13 (1, 3, 7). ILC2s are involved in immunity to extracellular parasites, and have important functions in tissue repair and regeneration (10, 11). ILC3s are characterized by expression of the RAR-related Orphan Receptor 2 (RORC2) and the production of IL-22, either alone or in combination with IL-17A and Granulocyte-Macrophage-Colony Stimulating Factor (GM-CSF) (1, 3, 7). They promote the defense against extracellular bacteria and fungi, and similar to ILC2s, can also participate in tissue repair processes (1, 12, 13). The majority of our current understanding of helper ILCs has come from murine studies. This is due in part to challenges in studying human ILCs that include; (i) non-NK cell ILCs are present in very low abundance in human blood (14), (ii) study of tissue-resident requires access to rare human samples, and (iii) a lack of tools to genetically manipulate human ILCs to explore molecular mechanism that control their development, function, and interactions with other immune and parenchymal cells.

CRISPR/Cas9 is an effective genome-editing tool that has emerged as a platform of choice for genetic manipulation (15). With CRISPR/Cas9 approaches, the endonuclease Cas9 induces a double-stranded break (DSB) in a specific target DNA sequence recognized by a guide RNA (gRNA) (15). The gRNA and Cas9 can be delivered into the cells via different approaches, including viral vector delivery and electroporation (16, 17). Those approaches can support gRNA and Cas9 being associated prior to delivery to form a ribonucleoprotein (RNP), or instead delivered in DNA form in a plasmid.

Previous studies have shown RNP approaches are associated with fewer off-target effects, reduced cytotoxicity (18) and increased genome editing efficiency (19). Lentiviral delivery requires CRISPR/Cas9 to be in a plasmid (DNA) format, and while very efficient, results in increased insertional mutagenesis and random integrations (16). Adenoviral (and adeno-associated) delivery also requires a DNA format, and although is non-integrating, is less efficient than lentiviral delivery. Adenoviral delivery also presents other disadvantages, such as limited cloning capacity and the potential for initiation of immune responses (16, 17). Electroporation-based delivery involves applying a high voltage pulse to cells that creates membrane pores, or nuclear pores in the case of nucleofection. It is generally reported to be as efficient as lentiviral delivery, and enables the use of both RNP and DNA formats, with reduced risks of mutagenesis (16). A caveat of this approach, however, is that it is often associated with poor cell viability (16). Thus when optimizing a CRISPR/Cas9 approach, it is important to consider cell viability, transfection efficiency and potential for off-target effects, and select an approach that balances these factors.

CRISPR/Cas9 has been used in multiple NK cells studies, providing protocols for NK cell lines (20), as well as primary human NK cells (21–27). Most human NK cells studies used electroporation delivery of an RNP (21–27), however, protocols differ in parameters such as electroporation settings and quantities of CRISPR/Cas9 components being delivered. Thus, even though this technique has been extensively used in NK cells, several factors could be optimized to enhance effectiveness of harnessing CRISPR/Cas9. Beyond NK cells, studies employing CRISPR/Cas9 in ILC2s or other ILCs are extremely limited, with to our knowledge only one protocol to date using plasmid delivery in mouse ILC2s (28, 29), and only one human study using lenti-CRISPR in human ILC2s to knockout PD-1 and HS3ST1 in stage IV colorectal patients ILC2s (30). Thus, development of CRISPR/Cas9 protocols are greatly needed to aid in translating mouse findings to human ILC2s, as well as to understand novel aspects of human ILC2s biology.

In this study, we developed an efficient protocol to mediate CRISPR/Cas9 knockout in human ILC2s. The protocol employed utilizes a nucleofection approach to deliver an RNP to knockout the cytokine IL-4, which was selected as a representative gene due to being a signature human ILC2 cytokine and an important mediator of ILC2s function (1). We report a method that achieves efficient and stable knockout of IL-4, while being optimized to minimize nucleofection-based impacts on cell viability. Having tested a wide range of parameters, the findings herein can aid development of protocols to target any gene of interest in human ILC2s.

## Materials and methods

### Human PBMC isolation

Healthy peripheral blood was obtained from donors through the Canadian Blood Services Blood4Research program, with each donor providing written, informed consent (UHN REB 17-6229, CBS Approved Study 2020-047). PBMCs were isolated using Lymphoprep (STEMCELL Technologies) per manufacturer instructions.

### ILC sorting and culture

Peripheral blood mononuclear cells (PBMCs) were stained with human TruStain FcX (BioLegend) and incubated with a cocktail of lineage antibodies conjugated to the FITC listed in **Supplemental Table 2**. Cells were washed in FACS buffer, resuspended in EasySep Buffer (STEMCELL Technologies), and enriched using the EasySep FITC Positive Selection Kit II (STEMCELL Technologies) per manufacturer instructions. Enriched cells were stained with antibody cocktail (**Supplemental Table 2**) and sorted using a FACSAria Fusion (BD Biosciences) or Symphony S6 sorter (BD Biosciences).

**Figure 1:**
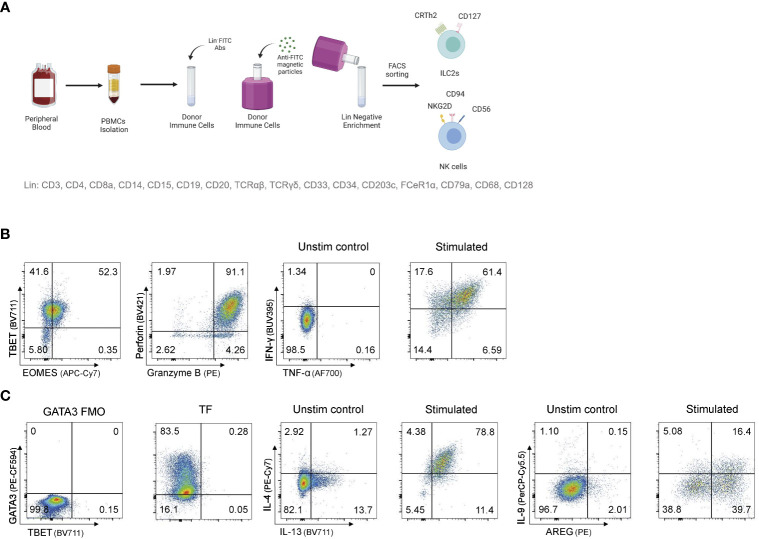
Sorting and expanding ILC2s and NK cells for CRISPR/Cas9 gene editing **(A)** Sorting strategy to isolate ILC2s and NK cells from human peripheral blood. PBMCs are stained for lineage with FITC antibodies and ILC2s and NK cells are FACS sorted after FITC^-^ PBMCs enrichment. **(B)** Representative TF expression and cytotoxic granules expression, and cytokine profile of NK cells after intracellular staining. **(C)** Representative TF expression and cytokine profile of ILC2s after intracellular staining. Figure in **(A)** created with Biorender.com.

PBMCs were sorted by flow cytometry as live, lineage negative populations. Specifically, CD56^dim^ NK cells were defined as live lineage^-^CD45^+^CD56^dim^CD16^+^, CD56^bright^ NK cells as live lineage^-^CD45^+^CD56^bright^CD16^-^ and ILC2s as live lineage^-^CD45^+^CD94^-^CD16^-^NKG2D^-^CD56^-^CD127^+^CRTh2^+^CCR6^-^. NK cells were cultured in MACS (Miltenyi) media supplemented with IL-2, IL-15, and IL-18 to maintain an activated state. Similarly, ILC2s were cultured in X-VIVO15 (Lonza) supplemented with 5% human AB serum (Sigma), 100U/mL Penicillin-Streptomycin (Gibco) and 1X GlutaMAX (Gibco) with recombinant human IL-2, IL-7, and IL-33 as described in Reid et al. (31). Cytokine analysis was performed pre and post IL-4 knockout by flow cytometry and cytometric bead array (**Figure 1**).

### Staining and flow cytometry

ILCs were counted and 100,000-200,000 cells were stained per condition. Cells were first washed with FACS buffer (PBS + 2% FCS) and blocked with human TruStain FcX (BioLegend) (diluted 1:10 in FACS buffer) for 15 min at 4°C. Next, they were stained for surface markers (**Supplemental Table 2**) (diluted in FACS buffer) for 30 min at 4°C, washed with FACS buffer, and fixed with FOXP3/Transcription Factor Staining set (eBioscience) buffer if performing phenotyping, or with 2% Paraformaldehyde (PFA) if assessing GFP expression. In the case of an intracellular staining, cells were washed with FACS buffer, and human TruStain FcX (BioLegend) (diluted 1:10 in permeabilization buffer from FOXP3/Transcription Factor Staining set (eBioscience)) was added one more time, 15 min at RT following surface staining and fixation. Cells were then stained for intracellular markers (**Supplemental Table 2**) in FOXP3/Transcription Factor Staining set permeabilization buffer (eBioscience) for 30 min at RT, then assessed by flow cytometry. Data was collected using a LSR Fortessa flow cytometer (BD Biosciences) and Diva software and analyzed with FlowJo v10.8.1 software.

### Cytokine stimulation

100,000-200,000 ILCs were plated in IL-2 overnight (O/N) the day before the stimulation. Supernatants were collected for Cytometric Bead Array (CBA). Cells were stimulated with PMA/Ionomycin (Invitrogen) for 6h. Golgi plug (BD Biosciences) and Golgi stop (BD Biosciences) were added after 4h. Following stimulation, cells were washed with FACS, and surface and intracellular flow cytometry staining was performed.

### Cytometric bead array

Cytometric bead array analysis was performed using the Legendplex Human Cytokine Panel kit (BioLegend) instructions. Briefly, 25 µL of supernatants collected after O/N incubation in IL-2 were transferred into a V-bottom plate. 25 µL of assay buffer and 25 µL of beads were also added. The plate was incubated for 2h while being shaken at 500 rpm. Wells were then washed twice and 25 µL of detection antibodies were added. The plate was incubated for 1h while being shaken at 500 rpm. Finally, 25 µL of Streptavidin-PE were added and the plate was incubated for 30 more minutes while being shaken at 500 rpm. Wells were then washed and read immediately by flow cytometry using a LSR Fortessa flow cytometer (BD Biosciences).

## Results

### Optimization of an electroporation-based CRISPR/Cas9 protocol for human ILC2s

As ILC2s are rare in blood, and access to tissue samples is limited, we elected to develop a protocol that could be used on activated and expanded human ILC2s, that for the purpose of this study were isolated from peripheral blood. Here, human ILC2s, as well as NK cells for a comparator, were isolated by flow cytometry sorting (**Figure 1A**), and then expanded in activating cytokines for 2 to 12 weeks. After expansion, NK cells and ILC2s maintained expression of signature cytokines and transcription factors (**Figures 1B, C**) allowing for testing gene knockout strategies on conventional ILC2-associated genes.

**Figure 2:**
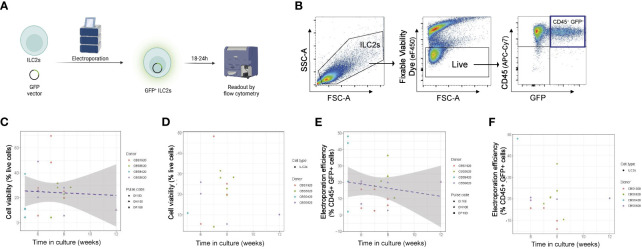
Optimization of time of ILC2 transfection across multiple electroporation settings. **(A)** ILC2s were transfected with a GFP vector by electroporation and GFP fluorescence was examined 18-24h post electroporation. **(B)** Representative gating of electroporated cells. The %live cells is referred to as viability and the %CD45^+^GFP^+^ cells is referred to as efficiency. **(C)** ILC2s viability in function of the time in culture across multiple pulse codes. **(D)** ILC2s viability in function of the time in culture for pulse code DN100. **(E)** Efficiency of ILC2s electroporation across multiple pulse codes. **(F)** Efficiency of ILC2s electroporation for pulse code DN100. (n=14-18). Figure in **(A)** created with Biorender.com.

In order to maximize genome targeting efficiency while reducing cytotoxicity and off-target editing (16, 18, 19), we elected to develop a CRISPR/Cas9 approach combining an RNP delivery format in combination with nucleofection (Lonza, **Supplemental Table 1**). This combination has been successfully employed in a wide range of studies in human T lymphocytes and NK cells (21–27, 32, 33), yet the major limitation is that it is often associated with poor viability (16). We therefore tested a wide range of nucleofector settings to identify nucleofector pulse codes that achieved the best efficiency possible while preserving cell viability (**Figure S1**; **Figure 2**). We conducted those experiments using a GFP vector (Lonza) as a marker for successful delivery of genetic material into the cells. Flow cytometry at 18-24h post-electroporation was used to assess efficiency, as this corresponded to peak GFP fluorescence expression (**Figure 2A**). Here, live CD45^+^GFP^+^ NK cells and ILC2s were assessed for a range of pulse codes (**Figure 2B**), and cell viability and electroporation efficiency (% CD45^+^GFP^+^ cells, gated on live) determined.

We noted that experiment-to-experiment, differences in the electroporation efficiency was observed in ILC2s from different donors. We hypothesized that, in addition to donor-to-donor variability, the time ILC2s were in culture prior to electroporation might influence the effectiveness of nucleofection. To assess this, we examined how both viability and efficiency of transfection across multiple pulse codes differed in experiments performed on NK cells and ILC2s expanded for different lengths of time *ex vivo* prior to transfection, and further assessed individually one of our lead pulse codes (DN100) **(** **Figures 2C-F**; **Figure S1** **)**. For both ILC2s and NK cells, cell viability post-electroporation was not impacted by the time in culture (**Figures 2C**; **S1D, E**, **B**), a finding that was observed across multiple pulse codes, including DN100 (**Figures 2C, D**; **S1D, E**; **S2B-E**). However, a negative correlation was clearly observed between electroporation efficiency and the time in culture for both ILC2s and NK cells, with an even stronger correlation in NK cells (**Figures 2E, F**; **S1F, G**; **S2F-I**). This trend was further confirmed when looking at individual pulse codes, including DN100 (**Figures 2F**; **S1F, G**; **S2G-I**). Therefore optimal nucleofection of both ILC2s and NK cells occurs with minimal *ex vivo* culturing time, with <6 weeks being acceptable for ILC2s and <5 weeks for NK cells.

**Figure 3:**
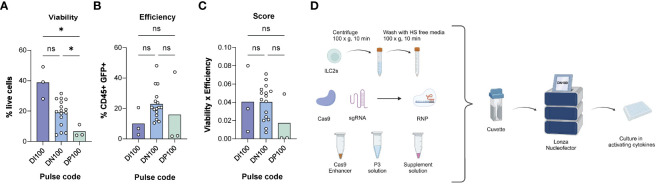
Determining the optimal electroporation pulse code for human ILC2s. **(A)** % live cells across pulse codes DI100, DN100 and DP100. **(B)** Efficiency of transfection across pulse codes DI100, DN100 and DP100. Efficiency was determined as the %CD45^+^GFP^+^ cells. **(C)** Score of pulse codes DI100, DN100 and DP100. Scores were calculated by multiplying %live cells x % CD45^+^GFP^+^ cells. **(D)** Optimized delivery protocol for CRISPR/Cas9 in ILC2s. 1M ILC2s are centrifuged at 100xg for 10 min and then washed with human serum (HS) free media. The pellet is then mixed with electroporation solutions and CRISPR/Cas9 in form of a RNP, and electroporated with the pulse code DN100. Following electroporation, cells are gently resuspended in their media overnight, and assessed for GFP expression at 24hrs (n=3-16). *= P ≤ 0.05, ns = non significant. Figure in **(D)** created with Biorender.com.

We next focused on defining the ideal nucleofector settings for human ILC2s. We first identified nucleofection codes previously used on primary human T cells (32, 33) or NK cells (21, 22, 24, 25), or recommended by the manufacturer. We then assessed cell viability and efficiency over a wide range of nucleofector settings (CA-137, CM-137, DH100, DI100, DN100, DP100, EH100, EN-138, EO-115, FI-115) (**Figures S1A-C**). Based on a preliminary screening, we identified the three lead pulse codes and performed multiple independent experiments using these codes to identify the nucleofector pulse code that would yield the greatest number of successfully transfected and viable ILC2s (**Figures 3A–C**). We examined nucleofection efficiency as well as cell viability and established an overall score that combined these parameters (multiplied cell viability by nucleofection efficiency). Throughout, NK cells were used as comparator (**Figures S2J-L**).

For these experiments 1x10^6^ ILC2s per condition were centrifuged at 100xg for 10 min at RT in 15 mL Falcon tubes. The supernatant was discarded, and cells were washed with 10 mL of serum free media to prevent any interference of the serum with CRISPR/Cas9 components. The cell pellet was mixed with electroporation solutions from the P3 primary cell kit (Lonza) as well as the GFP vector and transferred into a nucleocuvette. Next, ILC2s were electroporated using various pulses codes, immediately toped up with 80 µL warm complete media, and transferred into a 96-well plate pre-filled with their culture media.

Of nucleofector pulse codes tested, DI100 consistently maintained the highest ILC2 viability (38.70% ± 10.51), while DN100 had the highest efficiency (22.81% ± 10.09 CD45^+^GFP^+^ ILC2s). As a comparison, DN100 obtained the highest viability among pulse codes screened in NK cells (29.09% ± 14.78) and a similar efficiency to ILC2s (19.66% ± 14.54 CD45^+^GFP^+^ NK cells). When the combination of parameters was assessed, DI100 and DN100 obtained a similar score when used for ILC2s (0.04 ± 0.037 and 0.04 ± 0.020 respectively). However, DI100 efficiency was particularly low (10.0% ± 9.32 CD45^+^GFP^+^ cells), while DN100 maintained an acceptable viability (19.05% ± 8.65). Therefore, we moved forward to test additional parameters using DN100. An overview of the optimized RNP delivery protocol for CRISPR/Cas9 is detailed in **Figure 3D**. The next step was to determine an optimal RNP composition.

### Optimization of RNP composition for efficient IL-4 knockout

**Figure 4:**
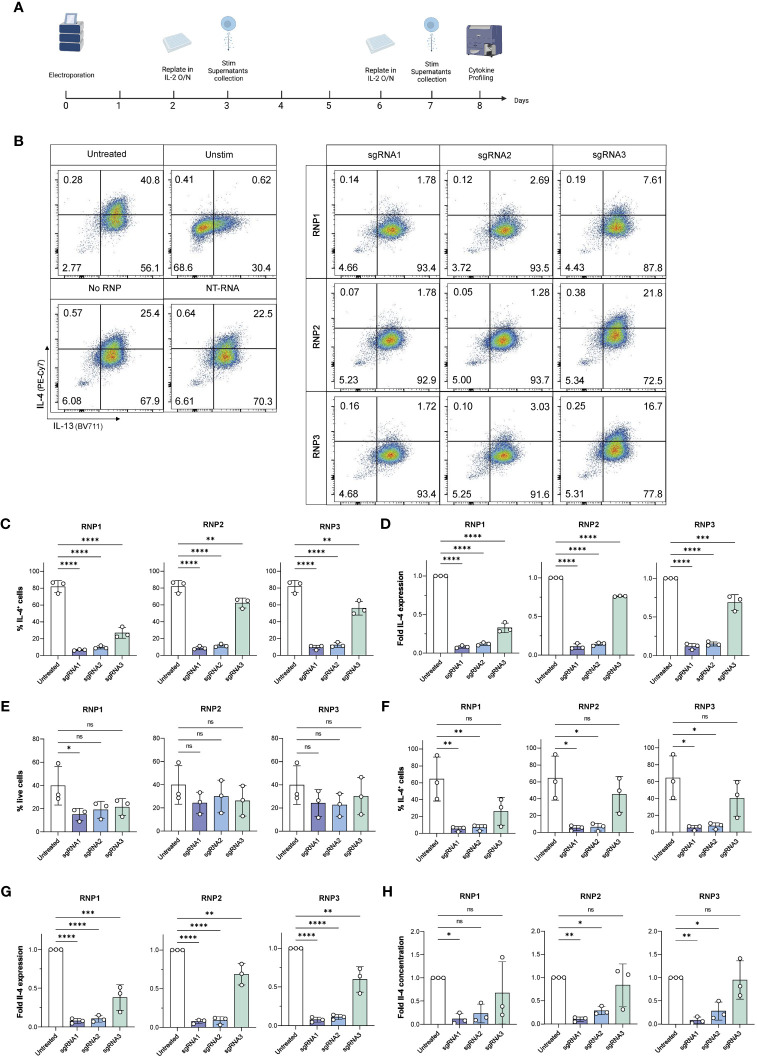
Viable, efficient and stable knockout of IL-4 in human ILC2s. **(A)** ILC2s were replated in IL-2 O/N at day 2 and day 6 following electroporation. At day 3 and day 7, supernatants were collected for Cytometric Bead Array analysis, and ILC2s were stimulated phorbol 12-myristate 13-acetate (PMA)/Ionomycin and stained intracellularly to examine their cytokine profile by flow cytometry. **(B)** Representative gating of IL-4 and IL-13 expression in untreated and knockout ILC2s at day 7. **(C)** Day 3 %IL-4^+^ cells in IL-4 knockout ILC2s electroporated with RNP Protocol 1, 2 or 3 (RNP1, RNP2, RNP3) compared to untreated ILC2sby flow cytometry after stimulation. **(D)** Day 3 fold change in IL-4 expression of IL-4 knockout ILC2s electroporated with RNP Protocol 1, 2 or 3 (RNP1, RNP2, RNP3) compared to untreated ILC2s by flow cytometry after stimulation. **(E)** Day 7 viability of IL-4 knockout ILC2s electroporated with RNP Protocol 1, 2 or 3 (RNP1, RNP2, RNP3) compared to untreated ILC2s by flow cytometry after stimulation. **(F)** Day 7 %IL-4^+^ cells in IL-4 knockout ILC2s electroporated with RNP Protocol 1, 2 or 3 (RNP1, RNP2, RNP3) compared to untreated ILC2s by flow cytometry after stimulation. **(G)** Day 7 fold change in IL-4 expression of IL-4 knockout ILC2s electroporated with RNP Protocol 1, 2 or 3 (RNP1, RNP2, RNP3) compared to untreated ILC2s by flow cytometry after stimulation. **(H)** Day 7 fold change in IL-4 concentration in supernatants of IL-4 knockout ILC2s electroporated with RNP Protocol 1, 2 or 3 (RNP1, RNP2, RNP3) compared to untreated ILC2s. (n=3). * = P ≤ 0.05; ** = P ≤ 0.01; *** = P ≤ 0.001; **** = P ≤ 0.0001, ns = non significant.

To determine optimal RNP composition, we elected to induce a CRISPR/Cas9 knockout of the cytokine IL-4, as it is stably expressed in high amounts by human ILC2s, making it an ideal proof-of-concept target. We tested three different RNP compositions (**Supplemental Table 3**) that differed in terms of sgRNA : Cas9 ratio, sgRNA and Cas9 quantities being delivered, and reconstitution buffer. For each RNP composition, we tested three different sgRNAs targeting *IL4* sequence (**Supplemental Table 4**). To determine our knockout efficiency, we assessed the IL-4 expression both by intracellular cytokine staining and measuring IL-4 secretion by cytometric bead array (CBA) of untreated ILC2s, control treated ILC2s or ILC2s receiving sgRNA targeting *IL4*. For IL-4 analysis by CBA, at day 2 and day 6 of ILC cultures, ILC2s were counted and replated in IL-2 overnight. Supernatants were then collected for subsequent secreted IL-4 analysis, as well as analysis of effects on other ILC-associated cytokines (**Figure 4A**; **Figure S3A**). Intracellular cytokine staining was performed on day 3 and day 7 post-transfection; the later time point being the most important, as our aim was to generate a protocol that results in stable genome edited cells to enable downstream *in vitro* or *in vivo* experiments. For intracellular cytokine staining, ILC2s were stimulated with PMA/Ionomycin and assessed for effective CRISPR/Cas9 knockout of IL-4, as well as other cytokines expressed by ILC2s or other ILC family members. A representative gating of IL-4 and IL-13 cytokines can be found in **Figure 4B**.

At day 3, ILC2s maintained good viability across all conditions, however RNP2 exhibited the highest viability across three independent donors (35.83% ± 14.35 – 39.83% ± 14.42 depending on the sgRNA used) (**Figure S3C**). When IL-4 expression was assessed in ILC2s, ILC2s receiving sgRNA1 and sgRNA2 displayed almost no IL-4 staining, with a fold IL-4 expression comprised between 0.08 and 0.12 for sgRNA1 and between 0.12 and 0.14 for sgRNA2, respectively, depending on the RNP (**Figures 4C, D**). We calculated the knockout score (% IL-4^+^ cells/% live cells), where a lower score meant a high knockout efficiency and a high viability. We observed sgRNA1 combined with RNP2 obtained the lowest score at day 3 (0.26 ± 0.04) (**Figure S3D**).

At day 7, no significant differences were observed in the viability of untreated and IL-4 ko ILC2s electroporated with the RNP2 and RNP3 protocol, regardless of the sgRNA used (**Figure 4E**). Both RNP2 and RNP3 did not have significant effects on cell viability. However, the RNP1 protocol had an overall lower viability, with a significant decrease when combining sgRNA1 and RNP1 (p = 0.042).

Both sgRNA1 and sgRNA2 effectively inhibited IL-4 expression in each RNP compositions tested (**Figure 4F**). The lowest IL-4 expression was achieved with sgRNA1 combined with RNP3 (5.12% ± 2.81 IL-4^+^ cells) followed by sgRNA1 combined with RNP2 (5.13% ± 10.09 3.07 IL-4^+^ cells) (**Figure 4F**). Accordingly, the fold decrease compared to untreated cells was most significant with sgRNA1 across all RNP protocols (p < 0.0001) (**Figure 4G**). The fold decrease compared to NT-RNA was also most significant with sgRNA1 across all RNP protocols (p < 0.0001) (**Figure S6I**). While sgRNA2 performed well, the combination of sgRNA1 with RNP2 obtained the best score (0.27 ± 0.25) (**Figure S4A**).

CBA analysis confirmed effective knockout of IL-4. We noted very low IL-4 secreted in ILC2s receiving with sgRNA1 across all donors compared to untreated ILC2s (5.74 ± 4.33 - 8.29 ± 7.33 pg/ml/100,000 cells for sgRNA1 transfected cells vs 97.05 ± 73.03 pg/ml/100,000 cells for untreated control) (**Figure S4B**). It is important to note that some of the individual repetitions were below the threshold of detection of the assay (2.83 pg/ml), meaning IL-4 was no longer detectable in those samples, and if above detection, was exceptionally low in the other independent experiments. The fold decrease in secreted IL-4 was significant with sgRNA1 across all RNPs, however the highest significance was observed with the combination of sgRNA1 and RNP2 (p = 0.004) (**Figure 4H**), in line with intracellular staining data for IL-4. While it is sometimes an effective strategy to combine two different sgRNAs, combining sgRNA1 and sgRNA2 did not result in better IL-4 knockout efficiency (**Figure S5**).

**Figure 5:**
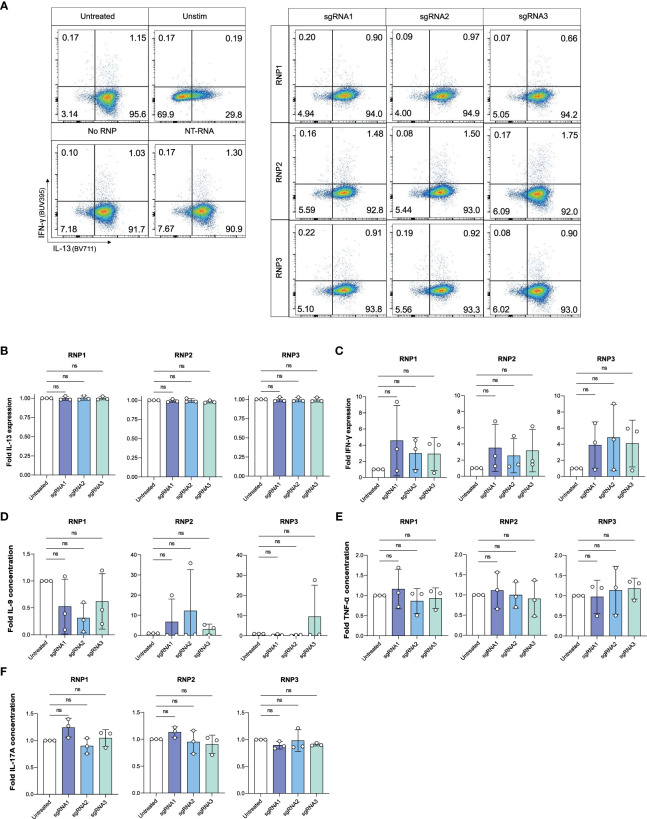
IL-4 knockout does not impact expression of other cytokines by ILC2s. **(A)** Representative gating of IFN-γ and IL-13 expression in untreated and knockout cells at Day 7. **(B)** Fold change in IL-13 expression of phorbol 12-myristate 13-acetate (PMA)/Ionomycin stimulated IL-4 knockout ILC2s electroporated with RNP Protocol 1, 2 or 3 (RNP1, RNP2, RNP3) or untreated ILC2s. **(C)** Fold change in IFN-γ expression of stimulated IL-4 knockout ILC2s electroporated with RNP Protocol 1, 2 or 3 (RNP1, RNP2, RNP3) or untreated ILC2s. **(D)** Fold change in IL-9 concentration in supernatants of IL-4 knockout ILC2s electroporated with RNP Protocol 1, 2 or 3 (RNP1, RNP2, RNP3) compared to untreated ILC2s. **(E)** Fold change in TNF-α concentration in supernatants of IL-4 knockout ILC2s electroporated with RNP Protocol 1, 2 or 3 (RNP1, RNP2, RNP3) compared to untreated ILC2s. **(F)** Fold change in IL-17A concentration in supernatants of IL-4 knockout ILC2s electroporated with RNP Protocol 1, 2 or 3 (RNP1, RNP2, RNP3) compared to untreated ILC2s. (n=3). ns = non significant.

To assess if CRISPR/Cas9 IL-4 knockout or nucleofection might have inadvertent effects on ILC2 functions, we examined if ILC2s receiving sgRNAs targeting the *IL4* sequence maintained expression of IL-13 and IL-9, two other ILC2-associated cytokines, or upregulated expression of cytokines associated with other ILCs such as IFN-γ or IL-17A (**Figure 5**). IL-13 expression was unchanged compared to untreated ILC2s (**Figures 5A, B**). Additionally, NK cell and ILC1 associated IFN-γ was not upregulated with IL-4 knockout (**Figures 5A, C**). When secreted cytokines were examined, expression of IL-9, TNF-α (associated with NK cells) and IL-17A (associated with ILC3s) were not altered in IL-4 knockout ILC2s (**Figures 5D–F**). Furthermore, cytokine expression (including IL-4) was not affected in non-targeting (NT) RNA and No RNP negative controls (**Figure S6**), further supporting the specificity of the IL-4 knockout.

To confirm our protocol would be effective at targeting other genes in ILC2s, we performed a pilot experiment with sgRNAs targeting *IL10*, which we and others have linked to ILC2s immunoregulatory effects in the context of allergy (11), cell therapy approaches for transplantation (28) and Graft-versus-Host Disease (GvHD) (31). We observed IL-10 expression was efficiently downregulated at day 3 post-transfection using our protocol (**Figure S7**), indicating the approach is effective at knocking out genes in different genomic positions.

**Figure 6:**
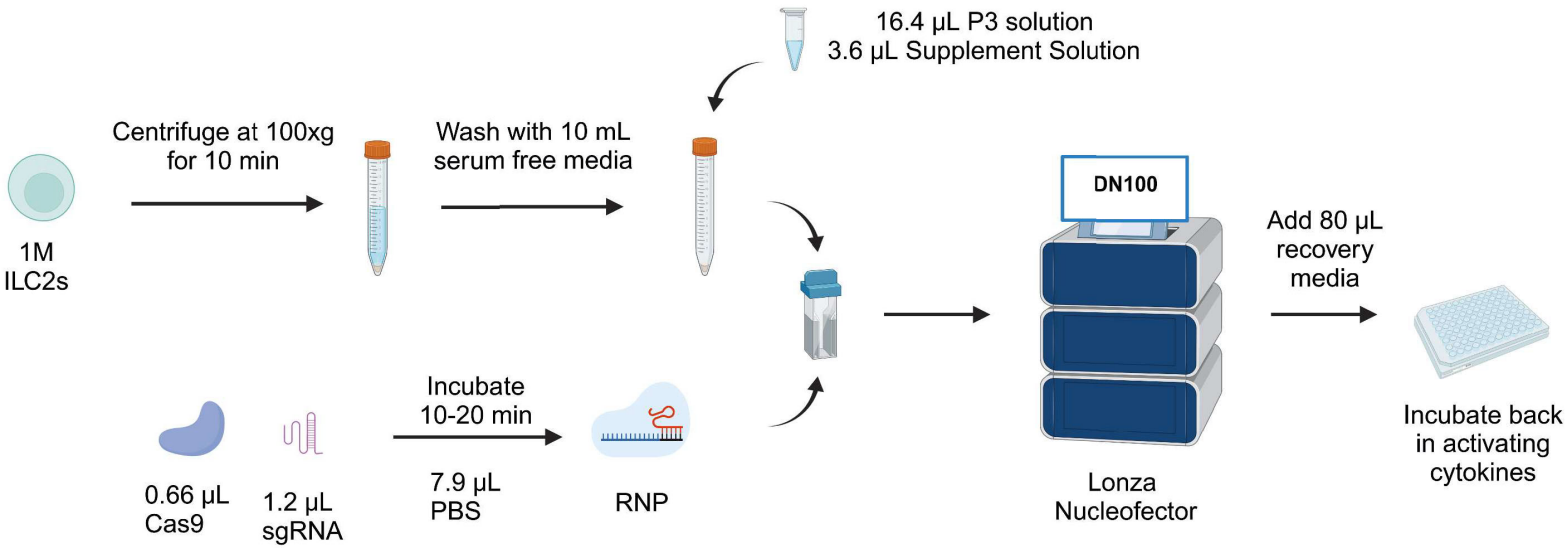
Workflow of CRISPR/Cas9-mediated knockout protocol in human ILC2s. ILC2s are centrifuged at 100xg for 10 min and washed with 10 mL serum free media. During that time, the RNP is reconstituted by mixing 1.2 µL sgRNA and 0.66 µL Cas9. The RNP is mixed with the pellet along with nucleofection solutions. Next, the mix is transferred into a nucleocuvette and electroporated with the pulse code DN100. Following electroporation, 80µL of recovery media is added and cells are incubated back in their activating cytokines. Figure created with Biorender.com.

Taken together, we report a protocol for efficient knockout of IL-4 in ILC2s that maintains good cell viability with sgRNA1 combined with RNP2, using the pulse code DN100. Day 7 post-knockout timepoint supports this result in stable IL-4 knockout. Furthermore, analysis of ILC2 and non-ILC2-associated cytokines supports CRISPR/Cas9 genome editing of IL-4 did not alter cytokine expression profiles of ILC2s. The overall protocol is summarized in **Figure 6**, with a complete detailed protocol included as **Supplemental Document 1**.

## Discussion

We report an effective CRISPR/Cas9 protocol to knockout genes of interest in human ILC2s. While IL-4 was used as a proof-of-concept target, optimization experiments included here can inform CRISPR/Cas9 gene editing strategies for targeting other genes of interest in human ILC2s. The nucleofection pulse code DN100 in combination with the RNP2 resulted in stable IL-4 knockout, and can be adapted to any target by screening for the best specific sgRNA for a gene of interest.

Here, CRISPR/Cas9 was delivered by electroporation, as this method has been proven successful in studies of human NK cells and T lymphocytes (21–27, 32–34). In addition, using an electroporation approach is associated with higher efficiency and less off-target effects in comparison to other techniques such as lentiviral delivery (16), and allowed us to deliver CRISPR/Cas9 in the form of an RNP. As expected, the main challenge associated with the electroporation was the poor viability. During the initial screening of pulse codes for ILC2s, we tested pulse codes previously used in human NK cells and T cells, as well as manufacturer’s pulse code recommendations for primary lymphocytes. Most of these settings led to efficient transfection in human NK cells, but resulted in either low viability or poor vector delivery to human ILC2s. While some pulse codes were associated with high viability, those generally displayed lower efficiency. On the other hand, pulse codes with a high percentage of CD45^+^GFP^+^ ILC2s had an extremely low percentage of live cells. For this reason, we established a score considering both parameters to assess the best pulse code. DN100, which had been successfully implemented in a study by Huang et al. in NK cells (27), appeared as an ideal middle ground for ILC2s, providing a relatively high efficiency while maintaining a reasonably high cell viability. We tried improving cell viability by letting ILC2s recover for 15 min the nucleocuvette in the incubator before transferring them into the 96-well plate, and by changing media 5 hours post-transfection, as previously documented (34), but this did not improve viability in ILC2s. Interestingly, electroporation seemed to be better tolerated by NK cells than helper ILCs. This is illustrated by consistent better viability and efficiency in NK cells with the same pulse codes. We also observed donor-to-donor variability in efficiency of GFP transfection in ILC2s, that is at least partially influenced by the length of time ILC2s were cultured, but also could be related to heterogeneity of ILC2s between donors. Of note, however, is that gene editing of *IL4* was less heterogeneous in terms of efficiency than transfection of the GFP vector.

We observed a very low percentage of IL-4^+^ cells when using sgRNA1 and sgRNA2. The efficiency of the knockout was better than anticipated, based on prediction with the GFP vector, with IL-4 expression in the knockout ILC2s reduced by approximately 10-fold, despite only achieved 25% transfection efficiency based on analysis of GFP expression. A possible explanation is the bigger size of the vector, which might prevent it to enter the pores as efficiently as the RNP. Indeed, it has been shown that a smaller sized-plasmid resulted better transfection efficiencies than a bigger sized-plasmid, suggesting the size of the format used to deliver CRISPR/Cas9 is an important parameter to consider (35). Furthermore, ILC2s viability post electroporation with the RNP was significantly higher than with the GFP vector. This could be explained by the fact that the GFP vector is delivered in a DNA form and thus needs to be transcribed and translated, exhausting the cell machinery, leading to higher cell death (17). In contrast, the RNP can directly reach the nucleus and induce the DSB without utilizing the cell machinery.

RNP composition 2 (RNP2), derived from Riggan et al. achieved the best viability (23). This is likely due to the difference in buffer used for the RNP reconstitution. RNP2 was reconstituted in PBS, whereas RNP1 and RNP3 were reconstituted in P3 nucleofection solution to reduce the volume being electroporated, which was another constraint in the protocol optimization. Reconstituting the RNP in PBS rather than nucleofection solution led to a higher cell viability, supporting that electroporation solution might be harmful to ILC2s when used for RNP reconstitution.

The choice of RNP did not impact IL-4 expression in controls, supporting that efficiency of knockout was sgRNA-dependent and not RNP-dependent. sgRNA1 and sgRNA2 outperformed sgRNA3, with sgRNA1 leading the highest editing efficiency. This was expected and concordant with the predicted scores. We tried to further optimize efficiency of the knockout by combing sgRNA1 and sgRNA2, however this did not improve the gene editing efficiency. CBA analysis of secreted cytokines confirmed the findings from intracellular cytokine staining. We noted that IL-4 expression tended as higher in NT-RNA and No RNP controls than in untreated cells by CBA (**Supplemental Figure 6**). The reason behind this is not completely clear, and may be due to the remaining cells after electroporation-induced cell death were the most viable and active. Another explanation is that the electroporation itself may activate ILC2s to a certain degree. While we analyzed our knockout compared to untreated controls as this is standard practice, knockout efficiency scores would have been even higher if compared to NT-RNA or No RNP controls (**Figure S6I**; **Supplemental Figure 6**).

We also assessed potential inadvertent impacts on ILC2s phenotype and cytokine expression with CRISPR/Cas9 IL-4 knockout. All parameters examined remained unchanged, beyond loss of IL-4 expression in IL-4 knockout ILC2s. However, a limitation of our study is that we did not perform extensive analysis of other potential off-target effects at the DNA level. For studies of ILC2 biology, this would be an important consideration, however analysis would differ between target genes of interest. For a given target gene, confirming that expression of nearby genes on the chromosome or that genes with similar sequences are not impacted is good practice.

While this protocol provides an efficient platform for knockout in human ILC2s, additional optimizations might improve CRISPR/Cas9 approaches. We observed a low percentage of IL-4^+^ cells even with sgRNA1 and RNP2. This low percentage is not the result of inefficient knock-down, as CRISPR/Cas9 acts directly on the cell DNA, but could rather be explained by transfection not being 100% efficient, and as a result not all the ILC2s being successfully transfected. In that regard, the purity of the sample could be increased by using a Cas9 coupled with GFP and sorting Cas9/GFP^+^ cells. In addition, CRISPR/Cas9 can sometimes induce silent mutations, as Non-Homologous End Joining (NHEJ) is a random process. As a result, some ILC2s could have been transfected but still express IL-4. In addition to increasing purity of knockout ILC2s, adapting this protocol (currently optimized for 1x10^6^ cells) to be able to transfect larger ILC2s numbers would be helpful for experiments requiring a higher yield. While it remains challenging to study human ILCs *ex vivo* due to the scarcity of human ILC2s in peripheral blood and limited tissue availability, more and more groups are developing efficient ways to expand human ILC2s to enable complex analysis of their biology, including assessment of effects on ILC2 metabolism, regulation, and *in vivo* function in humanized mice. Therefore, adapting this protocol to allow transfection of larger cell volumes or comparison with other approaches that preserve cell viability and do not present the disadvantages of viral vectors, such as peptide-mediated delivery (36), and viral-like particles (37) could be explored in future studies. The protocol we report herein, however, provides a base protocol for stable and efficient CRISPR/Cas9-mediated knockout of human ILC2s that maintains good cell viability, and can be employed to target any gene of interest in human ILC2s.

## Data availability statement

The original contributions presented in the study are included in the article/**Supplementary Material**. Further inquiries can be directed to the corresponding author.

## Ethics statement

The studies involving humans were approved by University Health Network Research Ethics Board. Healthy peripheral blood was obtained from donors through the Canadian Blood Services Blood4Research program, with each donor providing written, informed consent (UHN REB 17-6229, CBS Approved Study 2020-047).

## Author contributions

JA-C: Conceptualization, Data curation, Investigation, Methodology, Writing – original draft, Writing – review & editing, Formal Analysis, Validation, Visualization. JM: Investigation, Methodology, Writing – review & editing. SC: Investigation, Methodology, Writing – review & editing, Conceptualization, Data curation, Funding acquisition, Project administration, Resources, Software, Supervision, Writing – original draft.
