# Supplementary material for: Efficient and stable CRISPR/Cas9-mediated genome-editing of human type 2 innate lymphoid cells
Source: Front Immunol. 2023 Oct 5;14:1275413. doi: 10.3389/fimmu.2023.1275413 (PMC10585162; doi:10.3389/fimmu.2023.1275413)
Supplement: Supplementary file 1 [file DataSheet_1.pdf]

Efficient and stable CRISPR/Cas9-mediated genome-editing of human type 2 innate lymphoid cells

Johanne Audouze-Chaud[1,2], Jessica A. Mathews[2], and Sarah Q. Crome[1,2]

[1] Department of Immunology, Temerty Faculty of Medicine, University of Toronto, Toronto, Canada

[2] Toronto General Hospital Research Institute, Ajmera Transplant Centre, University Health Network, Toronto, Canada

ORCID identifiers:

SQC: 0000-0001-5117-7453

JAM: 0000-0003-2926-8222

***Correspondence:**

Sarah Q. Crome

email: sarah.crome@utoronto.ca

phone: 416.634.8097

Keywords: innate lymphoid cells, ILC2s, Natural Killer cells, CRISPR/Cas9

Supplemental Tables

Supplemental Table 1: Material and Equipment

Equipment	Catalog #	Company
4D Nucleofector Core Unit	AAF-1003B	Lonza
4D Nucleofector X Unit	AAF-1003X	Lonza
SF Cell Line 4D-Nucleofector™ X Kit S	V4XC-2032	Lonza

Supplemental Table 2: List of antibodies

Epitope	Fluorophore	Company	Clone	Dilution
CD3	FITC	BioLegend	OKT3	1:100
CD3	FITC	BioLegend	UCHT1	1:100
CD4	FITC	BioLegend	RPAT4	1:200
CD8a	FITC	BioLegend	RPAT8	1:200
CD14	FITC	BioLegend	M5E2	1:200
CD15	FITC	BioLegend	W6D3	1:200
CD19	FITC	BioLegend	HIB19	1:200
CD20	FITC	BioLegend	2H7	1:200
TCRab	FITC	BioLegend	IP26	1:200
TCRgd	FITC	BioLegend	B1	1:200
CD33	FITC	BioLegend	HIM3-4	1:100
CD34	FITC	BioLegend	581	1:100
CD203c	FITC	BioLegend	NP4D6	1:100
FceR1a	FITC	BioLegend	AER37	1:100
CD79a	FITC	BioLegend	HM47	1:100
CD138	FITC	BioLegend	MI15	1:100
CD94	PerCP/Cy5.5	BioLegend	DX22	1:100
NKG2D	PerCP/Cy5.5	BioLegend	1D11	1:100
CD127	PE	BD	HIL-7R-M21	1:50
CD16	PE-Dazzle594	BioLegend	3G8	1:100
CD117	PE/Cy7	BioLegend	1D11	1:50
CCR6	APC	BioLegend	G034e3	1:25
Fixable Viability Dye	AlexaFluor700	BD Biosciences		1:1000
CD45	APC/Cy7	BioLegend	HI30	1:100
CRTh2	BV421	BioLegend	BM16 (RUO)	1:25

CD56 (NCAM)	BV605	BioLegend	HCD56	1:50
CD16	BUV737	BD Biosciences	3G8	1:100
Fixable Viability Dye	eF506	BioLegend	-	1:800
IL-10	Alexa488	Invitrogen	JES3-9D7	1:25
IL-9	PerCP/eFluor710	Invitrogen	MH9D1	1:50
Amphiregulin	PE	Invitrogen	AREG559	1:25
IL-17F	PE-CF594	BD Biosciences	O33-782	1:50
IL-4	PE-Cy7	BioLegend	MP4-25D2	1:50
IFNg	BUV395	BD Biosciences	B27 (RUO)	1:100
GM-CSF	APC	BioLegend	BVD2-21C11	1:50
TNFa	Alexa700	eBioscience	MAB11	1:50
IL-22	eF450	eBioscience	22URTI	1:50
IL-17A	BV786	BD Biosciences	N49-653	1:50
IL-13	BV711	BD Biosciences	JES10-5A2	1:50
IL-2	BV650	BD Biosciences	5344.111	1:50
IL-10	PE	Invitrogen	JES3-9D7	1:50
TBET	BV711	BioLegend	4B10	1:50
EOMES	APC-Cy7	Invitrogen	WD1928	1:50
GATA3	PE-CF594	BD Bioscience	L50-823	1:50
Perforin	BV421	Invitrogen	dG9	1:50
Granzyme B	PE	eBioscience	GB11	1:50

Supplemental Table 3: RNP compositions

	**RNP1**	**RNP2**	**RNP3**
Buffer	P3 – 6 µL	PBS – 7.9 µL	P3 – 7.9 µL
sgRNA	3.43 µL<br>343 pmol	1.2 µL<br>120 pmol	1.2 µL<br>120 pmol
Cas9	4.86 µL<br>297 pmol	0.66 µL<br>40 pmol	0.66 µL<br>40 pmol

Supplemental Table 4: Sequences of IL-4 targeting sgRNAs

sgRNA reference	Strand	Sequence	PAM	On-target score	Off-target score
Hs.Cas9.IL4.1.AA (sgRNA1)	-	GTGTCCGTGGACAAAGTTGC	CGG	65	81
Hs.Cas9.IL4.1.AB (sgRNA2)	+	CAAGTGCGATATCACCTTAC	AGG	72	80
Hs.Cas9.IL4.1.AC (sgRNA3)	+	TGAGAAGGACACTCGCTGCC	TGG	52	68

Supplemental Table 5: Volumes of IL-4 ko conditions

Condition	P3 solution (µL)	Supplement (µL)	RNP (µL)	Enhancer (µL)
RNP1	9.25	3.6	14.3	2.86
RNP2	16.4	3.6	9.76	0.9
RNP3	8.5	3.6	9.76	0.9
NT-RNA	16.4	3.6	9.76	0.9
No RNP	16.4	3.6	-	-

Supplemental Table 6: Sequences of IL-10 targeting sgRNAs

sgRNA reference	Strand	Sequence	PAM	On-target score	Off-target score
Hs.Cas9.IL10.1.AA (sgRNA1)	-	GTTGTTAAAGGAGTCCTTGC	TGG	79	57
Hs.Cas9.IL10.1.AB (sgRNA2)	+	GGATCATCTCAGACAAGGCT	TGG	64	43
CD.Cas9.JNBB1600.AQ (sgRNA3)	-	TCGTATCTTCATTGTCATGT	AGG	53	53

Supplemental Figures

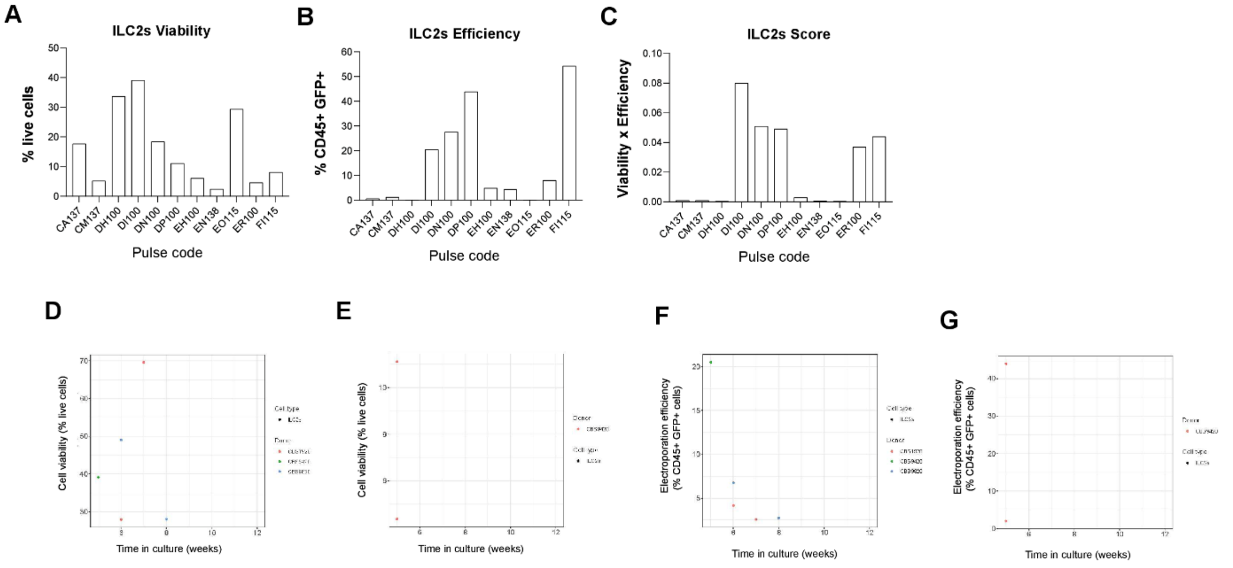

Supplemental Figure 1: Initial pulse code screening and determination of optimal electroporation time for ILC2s. (A) Initial screening of lLC2s viability for different pulse codes. **(B)** Initial screening of efficiency (% $CD45^+GFP^+$ cells) of different pulse codes in ILC2s. **(C)** Initial screening of scores of different pulse codes in ILC2s. Scores were calculated by multiplying %live cells x % $CD45^+GFP^+$ cells. **(D)** ILC2s viability in function of the time in culture for pulse code DI100. **(E)** ILC2s viability in function of the time in culture for pulse code DP100. **(F)** Efficiency of ILC2s electroporation for pulse code DI100. **(G)** Efficiency of ILC2s electroporation for pulse code DP100. (n=2-5)

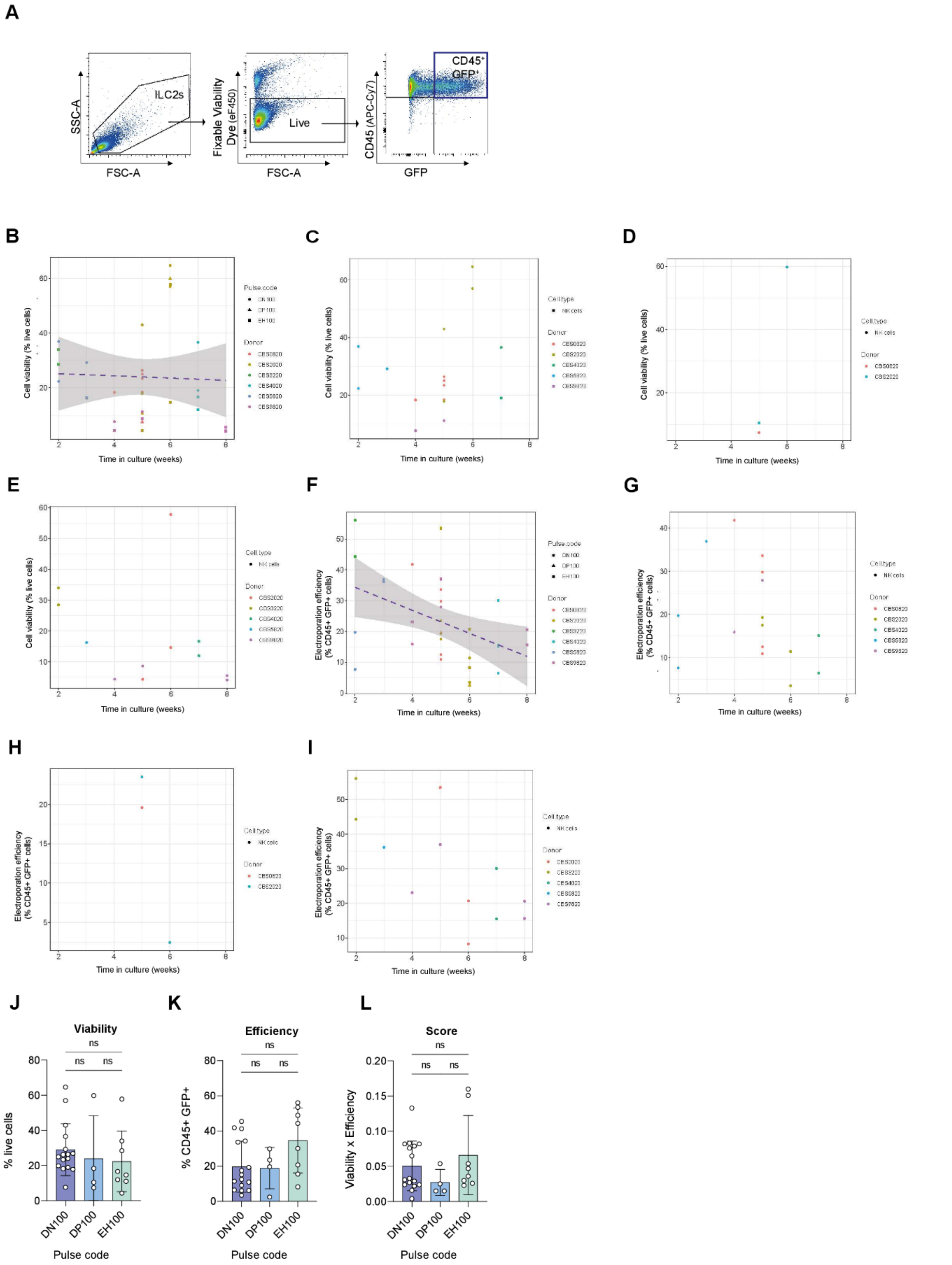

Supplemental Figure 2: Determination of optimal electroporation time and settings for NK cells. (A) Representative gating of electroporated cells. The %live cells is referred to as viability and the %CD45^{+}GFP^{+} cells is referred to as efficiency. **(B)** NK cells viability in function of the time in culture across multiple pulse codes. **(C, D, E)** ILC2s viability in function

of the time in culture for pulse codes DN100, DI100 and DP100. **(F)** Efficiency of ILC2s electroporation across multiple pulse codes. **(G, H, I)** Efficiency of ILC2s electroporation for pulse codes DN100, DI100 and DP100. **(J)** % live cells across pulse codes DN100, DP100 and EH100. **(K)** Efficiency of transfection across pulse codes DN100, DP100 and EH100. Efficiency was determined as the %$CD45^{+}GFP^{+}$ cells. **(L)** Score of pulse codes DN100, DP100 and EH100. Scores were calculated by multiplying %live cells x % $CD45^{+}GFP^{+}$ cells. (n=4-16)

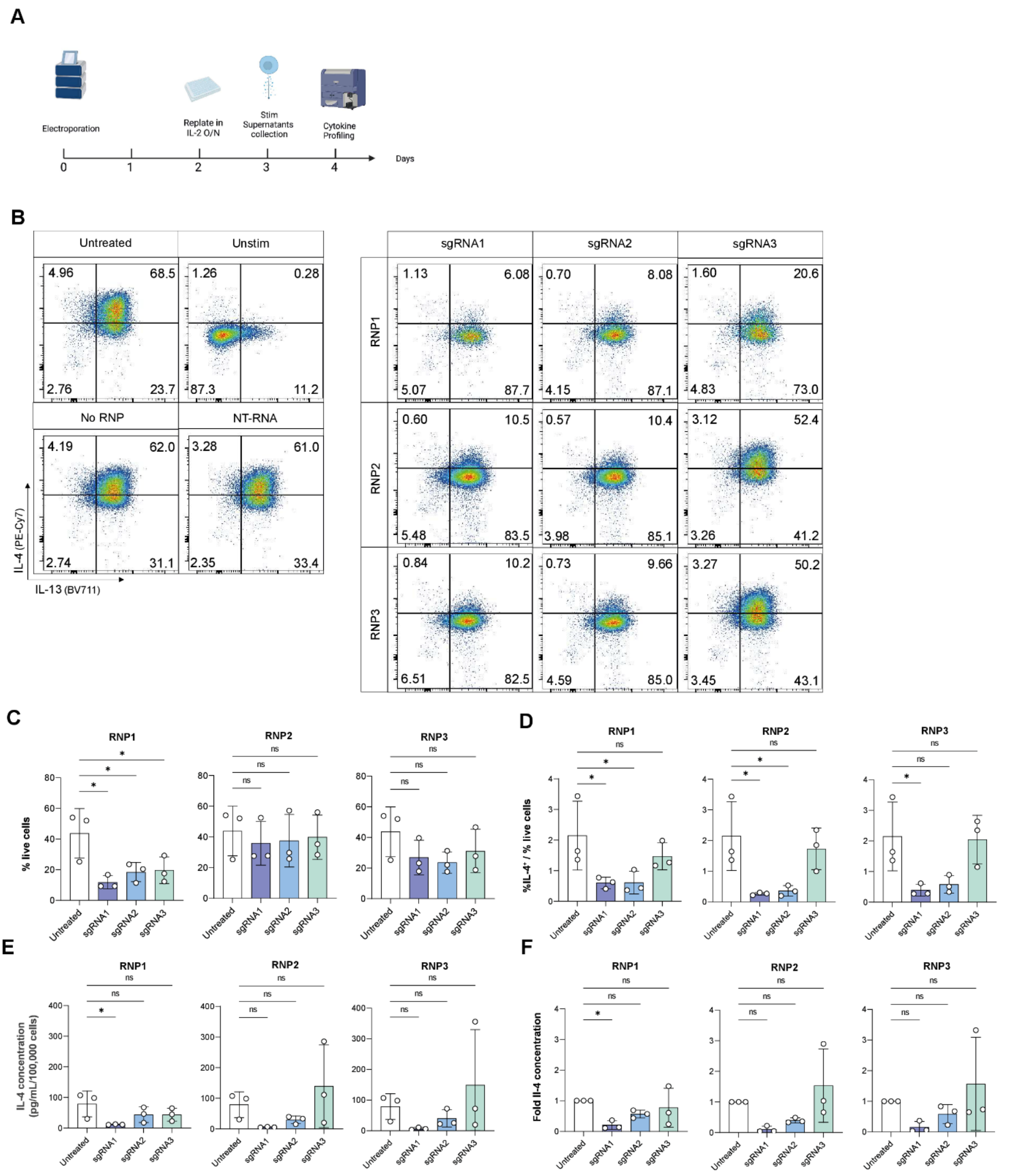

Supplemental Figure 3: Assessment of viiability, efficiency and IL-4 knockout stability in human ILC2s at day 3 post-electroporation. (A) ILC2s were replated in IL-2 O/N at day 2 following electroporation. At day 3, supernatants were collected and run by Cytometric Bead Array, and ILC2s were stimulated phorbol 12-myristate 13-acetate (PMA)/Ionomycin and stained intracellularly to examine their cytokine profile by flow cytometry. **(B)** Representative gating of IL-4 and IL-13 expression in untreated and knockout ILC2s. **(C)** Viability of IL-4 knockout ILC2selectroporated with RNP Protocol 1, 2 or 3 (RNP1, RNP2, RNP3) compared to untreated ILC2s by flow cytometry after stimulation. **(D)** Score of IL-4 knockout ILC2s electroporated with RNP Protocol 1, 2 or 3 (RNP1, RNP2, RNP3) compared to untreated ILC2s.

Scores were calculated as %IL-4^+ cells / %live cells. **(E)** IL-4 concentration in supernatants of IL-4 knockout ILC2s electroporated with RNP Protocol 1, 2 or 3 (RNP1, RNP2, RNP3) compared to untreated ILC2s. **(F)** Fold change in IL-4 concentration in supernatants of IL-4 knockout ILC2s electroporated with RNP Protocol 1, 2 or 3 (RNP1, RNP2, RNP3) compared to untreated ILC2s. (n=3)

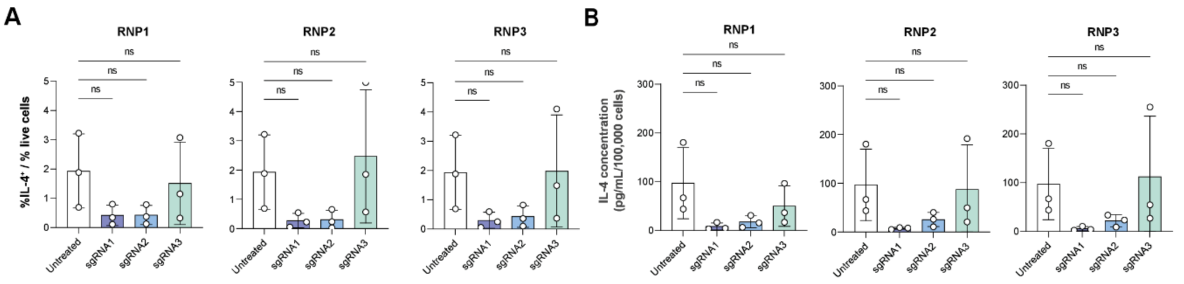

Supplemental Figure 4: sgRNA1 combined with RNP2 resulted in optimal IL-4 knockout. **(A)** Score of IL-4 knockout ILC2s electroporated with RNP Protocol 1, 2 or 3 (RNP1, RNP2, RNP3) compared to untreated ILC2s at day 7. Scores were calculated as %IL-4^{+} cells / %live ILC2s. **(B)** IL-4 concentration in supernatants of IL-4 knockout ILC2s electroporated with RNP Protocol 1, 2 or 3 (RNP1, RNP2, RNP3) compared to untreated ILC2s at day 7.

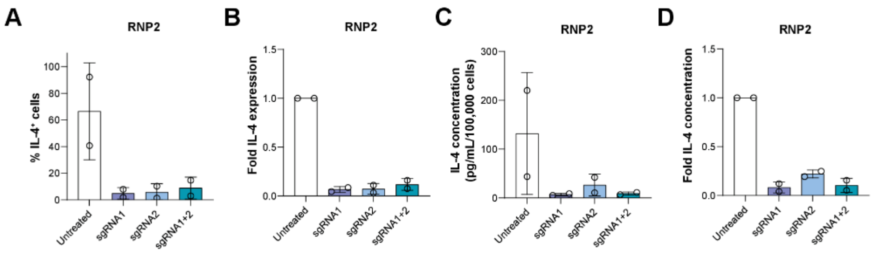

Supplemental Figure 5: Combining sgRNA1 and sgRNA2 does not improve knockout efficiency. (A) Day 7 %IL-4^{+} cells in IL-4 knockout ILC2s electroporated with RNP Protocol 2 (RNP2) using either sgRNA1, sgRNA2, or a combination of sgRNA1+sgRNA2. **(B)** Day 7 fold change in IL-4 expression of IL-4 knockout ILC2s electroporated with RNP Protocol 2 (RNP2) using either sgRNA1, sgRNA2, or a combination of sgRNA1+sgRNA2, compared to untreated ILC2s by flow cytometry after stimulation. **(C)** Day 7 IL-4 concentration of IL-4 knockout ILC2s electroporated with RNP Protocol 2 (RNP2), using either sgRNA1, sgRNA2, or a combination of sgRNA1+sgRNA2, measured by Cytometric Bead Array. **(D)** Day 7 fold change in IL-4 concentration in supernatants of IL-4 knockout ILC2s electroporated with RNP Protocol 2 (RNP2), using either sgRNA1, sgRNA2, or a combination of sgRNA1+sgRNA2, compared to untreated ILC2s. (n=2)

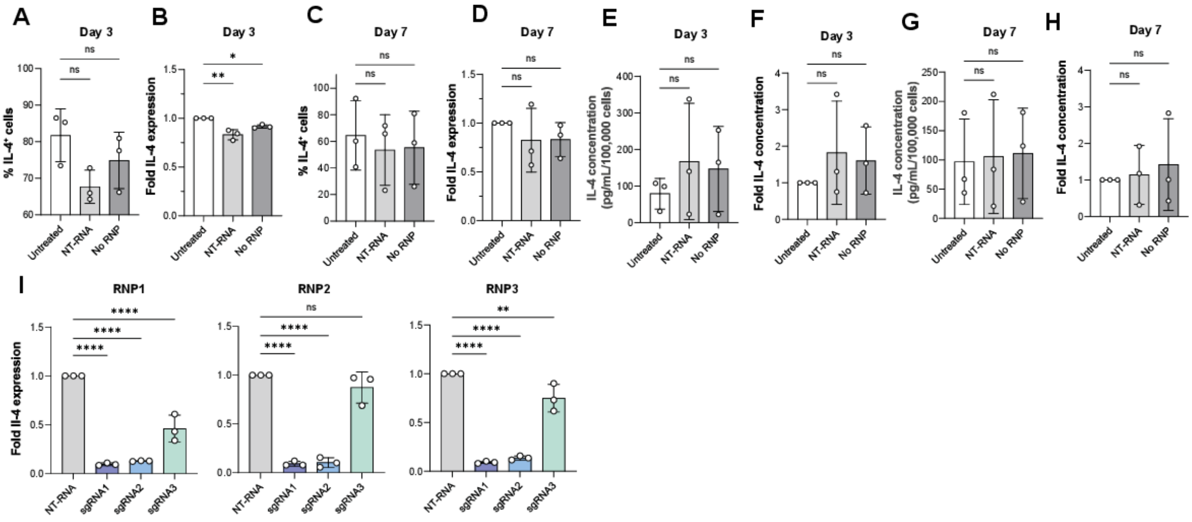

Supplemental Figure 6: Electroporation itself does not impact IL-4 expression. Along untreated ILC2s, ILC2s receiving Non-Targeting (NT)-RNA or No RNP were included as controls. NT-RNA contains a sequence binding to a random site of the genome and should not result in any knockout. No RNP ILC2s are electroporated without any RNP. **(A)** %IL-4^{+} cells in IL-4 knockout cells after stimulation at day 3 post-electroporation. **(B)** Fold change in IL-4 expression of IL-4 knockout ILC2s after stimulation at day 3 post-electroporation. **(C)** %IL-4^{+} cells in IL-4 knockout ILC2s after stimulation at day 7 post-electroporation. **(D)** Fold change in IL-4 expression of IL-4 knockout ILC2s after stimulation at day 7 post-electroporation. **(E)** IL-4 concentration in supernatants of IL-4 knockout ILC2s at day 3. **(F)** Fold change in IL-4 concentration in supernatants of IL-4 knockout ILC2s at day 3. **(G)** IL-4 concentration in supernatants of IL-4 knockout ILC2s at day 7. **(H)** Fold change in IL-4 concentration in supernatants of IL-4 knockout ILC2s at day 7. **(I)** Day 7 fold change in IL-4 expression of IL-4 knockout ILC2s electroporated with RNP Protocol 1, 2 or 3 (RNP1, RNP2, RNP3) compared to ILC2s electroporated with NT-RNA by flow cytometry after stimulation. (n=3)

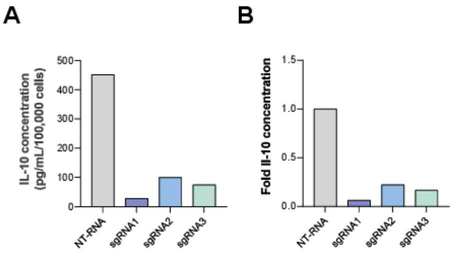

Supplemental Figure 7: CRISPR/Cas9 protocol also effective for knockout of IL-10. IL-10 was targeted in ILC2s using the optimized knockout protocol to confirm effectiveness at knocking out additional genes of interest in ILC2s. The efficiency of the knockout was assessed at Day 3. (A) IL-10 concentration in supernatants of IL-10 knockout ILC2s at Day 3, compared to ILC2s receiving non-targeting (NT) RNA. (B) Fold change in IL-10 concentration in supernatants of IL-10 knockout ILC2s at Day 3 compared to NT RNA control (n=1).

Supplemental Document 1: SOP for CRISPR/Cas9-mediated knockout in human ILC2s

1. Count cells: ___
   Conditions : ___ sgRNA(s), NT-RNA, No RNP
   ___ conditions = ____ M cells needed.
2. Prepare a 96-well plate with complete cytokine media at the same concentrations used for ILC2s culture and prewarm in the incubator (3 wells per condition). Prewarm 80 µL complete media (without cytokines) per condition. This will be used as recovery media once the cells have been transfected.
   ___ conditions x 80 = ____ µL recovery media
3. Transfer 1M ILC2s per condition into a 15 mL Falcon tube.
   Conditions : ___ sgRNA(s), NT-RNA, No RNP
   ___ conditions = ____ M cells → ____ mL
4. Centrifuge ILC2s at 100xg for 10 min. Discard supernatant.
5. Wash with 10 mL serum-free media at 100xg for 10 min. Discard supernatant – no liquid should be left on top of the pellet.
6. During the centrifugations, prepare the RNP. For 1M cells, mix 7.9 µL sterile PBS, 1.2 µL (120 pmol) sgRNA (or NT-RNA) and 0.66 µL (40 pmol) Cas9. Prepare each RNP in individual microcentrifuge tubes.
   Incubate 10-20 min at RT.
7. After discarding supernatant from the cells, add 16.4 µL P3 solution and 3.6 µL Supplement solution into the tube. Mix gently.
   ___ M cells → ___ x 16.4 = ___ µL P3 solution + ___ x 3.6 = ___ µL Supplement solution
   Mix gently (pipette up and down 3 times maximum).
8. Take 20 µL of the cell mix and add it into the microcentrifuge tubes containing the RNP mix. There will be 20 µL left in the Falcon tube, which correspond to the No RNP control.
9. Add 0.9 µL Cas9 enhancer to each microcentrifuge tube.
10. Transfer the cell-RNP mix (30 µL) into the nucleocuvettes (Lonza).
11. Electroporate with the pulse code DN100.
12. Add 80 µL prewarmed recovery media per nucleocuvette by pouring it gently on the side of the cuvette.
13. Mix gently 2-3 times and add 40 µL of transfected cells per well.
14. Change media every other day until readout.
